# Cadmium Handling, Toxicity and Molecular Targets Involved during Pregnancy: Lessons from Experimental Models

**DOI:** 10.3390/ijms18071590

**Published:** 2017-07-22

**Authors:** Tania Jacobo-Estrada, Mitzi Santoyo-Sánchez, Frank Thévenod, Olivier Barbier

**Affiliations:** 1 Departamento de Sociedad y Política Ambiental, CIIEMAD, Instituto Politécnico Nacional, 30 de Junio de 1520 s/n, La Laguna Ticomán, Ciudad de México 07340, Mexico; tjacoboe@ipn.mx; 2 Departamento de Toxicología, Centro de Investigación y de Estudios Avanzados del Instituto Politécnico Nacional, Av. Instituto Politécnico Nacional 2508, Gustavo A. Madero, San Pedro Zacatenco, Ciudad de México 07360, Mexico; santoyomitzi@gmail.com; 3 Department of Physiology, Pathophysiology & Toxicology and ZBAF (Centre for Biomedical Education and Research), Faculty of Health-School of Medicine, Witten/Herdecke University, Stockumer Str 12 (Thyssenhaus), D 58453 Witten, Germany; frank.thevenod@uni-wh.de

**Keywords:** cadmium, pregnancy, multiple organs toxicity, fetus, placenta

## Abstract

Even decades after the discovery of Cadmium (Cd) toxicity, research on this heavy metal is still a hot topic in scientific literature: as we wrote this review, more than 1440 scientific articles had been published and listed by the PubMed.gov website during 2017. Cadmium is one of the most common and harmful heavy metals present in our environment. Since pregnancy is a very particular physiological condition that could impact and modify essential pathways involved in the handling of Cd, the prenatal life is a critical stage for exposure to this non-essential element. To give the reader an overview of the possible mechanisms involved in the multiple organ toxic effects in fetuses after the exposure to Cd during pregnancy, we decided to compile some of the most relevant experimental studies performed in experimental models and to summarize the advances in this field such as the Cd distribution and the factors that could alter it (diet, binding-proteins and membrane transporters), the Cd-induced toxicity in dams (preeclampsia, fertility, kidney injury, alteration in essential element homeostasis and bone mineralization), in placenta and in fetus (teratogenicity, central nervous system, liver and kidney).

## 1. Introduction

Cadmium (Cd) is one of the most common and harmful transition metals present in our environment. Unfortunately, this non-essential element is toxic at very low doses and non-biodegradable with a very long biological half-life. Kjellström and Nordberg were the first researchers to work on long term persistence of Cd body loads and to evaluate the half-life of Cd, establishing a range of half-times from 6 to 38 years for the human kidney and 4 to 19 years for the human liver [1].

Whereas the molecular and cellular mechanisms of Cd toxicity are studied in great detail, as well as the toxicokinetics and toxicodynamics of Cd [2], many other aspects need to be elucidated considering the impact of Cd exposure. Numerous parameters are crucial to consider during the risk assessment of toxicants and environmental pollutants since they could intrinsically modify the absorption, distribution, metabolism, and excretion of these xenobiotics by the host organism. Hence, the genetic background, the epigenetic modifications, the diet, a physio-pathological condition, the individual behavior, and the co-exposure to other xenobiotics must be considered [3,4].

A unique physiological condition that could impact and modify essential pathways involved in the handling of Cd (and other environmental pollutants) is pregnancy [5,6]. With regard to the mother, during gestation, most organs and systems (renal, cardiovascular, blood, gastrointestinal, respiratory and endocrine) show multi-faceted and progressive changes in their anatomy and functions; e.g., ventilation is significantly increased (amplifying the contact with airborne molecules and particulate matter), total body water and plasma volume increase (modifying the volume of distribution and lowering the concentrations of proteins and elements in the body fluids), glomerular filtration rate increases (accelerating the proximal uptake of small filtered binding-proteins and/or their excretion through urine), gastrointestinal motility decreases whereas gastric pH increases (modifying the absorption), etc. [6]. Added to this situation, it is also necessary to consider the appearance of a new and temporary organ, the placenta, whose function participates importantly in the specific toxicity towards the fetus. If the placenta regulates blood flow, plays the role of a transport barrier, and metabolizes chemicals, it can also be a target for xenobiotics-induced toxicity [5,7,8]. Regarding the fetus, in utero/prenatal life corresponds to a challenging period due the rapid changes that occur during development. From preimplantation to organogenesis and finally birth, molecular expression, cell differentiation, structures and functions of the new organs undergo profound and dynamic modifications [9]. Thus, the nature of the fetus as a target of toxicity and the molecular pathways involved are constantly changing too [5,10].

Over the years, epidemiological studies have demonstrated the critical role of prenatal exposure to cadmium in the development of human individuals and its impact on public health. Thus, birth outcomes related to Cd exposure, such as alteration of Apgar 5-min score, birth weight, risk of being small-for-gestational age [11,12,13], association with poorer cognition [14] or epigenetic modifications [15,16], among others, have been evidenced in populations from all over the world.

To give the reader an overview of the possible mechanisms involved in the multiple organ toxic effects in fetuses and dams after exposure to Cd during pregnancy, we decided to compile some of the most relevant experimental studies and summarize the advances in this field during the last decades.

## 2. General Information about Cd

### 2.1. Mode of Action (MOA) of Cd Toxicity

As reviewed before [17,18,19,20,21], the toxic form of Cd is the ionized form Cd^2+^. Conjugates and bound forms of filtered Cd are not toxic by themselves but the divalent form released from the complexes is responsible for the cellular toxicity altering the mitochondrial activity (through outer membrane rupture and uncoupling of respiration), provoking oxidative stress and apoptosis, interacting with transporter and ion channels, among others. Cadmium and divalent metals are well-known inducers of Ca^2+^ mobilization with a potency order of Cd^2+^ > Co^2+^ Ni^2+^ > Fe^2+^ > Mn^2+^ [22]. Moreover, Cd^2+^ can compete with Zn^2+^ and Ca^2+^ transport and alters the uptake and cellular homeostasis of these important ligands, enzyme-cofactor and second messenger [23]. Thus, Cd^2+^ can be responsible for interfering with Ca^2+^ and Zn^2+^ pathways and, consequently, disrupting signaling, transport, metabolism and cell fate [24].

The main MOA of Cd toxicity is the induction of cell death through oxidative stress [25]. Ca^2+^ and reactive oxygen species (ROS), which are molecular triggers controlling cell function and activate effectors (Apoptosis signal-regulating kinase 1-c-Jun N-terminal kinase/p38, calpains, caspases and ceramides), are also capable to cause irreversible damages to organelles, such as mitochondria and endoplasmic reticulum (ER). Interestingly, localized ROS/Ca^2+^ levels play a role as second messengers triggering mechanisms such as cellular adaptation and survival via the signal transduction (extracellular signal-regulated kinases-1/2, phosphoinositide-3-kinase–protein kinase AKt) and the transcriptional regulation (redox effector factor-1 Ref1- nuclear factor erythroid 2-related factor 2 Nrf2, nuclear factor kappa-light-chain-enhancer of activated B cells NF-κB, wnt, activator protein 1 AP-1, bestrophin-3). Of course, many different proteins and processes mediated by ROS/Ca^2+^ signaling (metallothionein, B-cell lymphoma 2 proteins, ubiquitin-proteasome, ER stress associated with unfolded proteins, autophagy, cell cycle regulation) can induce dual responses including death or survival, in accordance to the cell type and the conditions of exposure [20,24].

### 2.2. Sources of Exposure

Cadmium is a transition metal (group II b) with eight stable isotopes that was discovered by the German chemist Strohmeier in 1817, because of the study of some of the impurities of zinc carbonate [26].

This metal is found in nature in many forms. The most common are: elemental cadmium (Cd^0^), and as cadmium carbonate, chloride, oxide, sulfate and sulfide salts. Considering natural sources, Cd is widely distributed in the earth’s crust. Rock wear and erosion result in the release of Cd. Volcanic activity on marine and terrestrial surfaces also contributes to its release [27]. Considering anthropogenic sources, Cd is released from human-related activities. Cadmium is generally obtained as a by-product of the zinc concentrates. The zinc: cadmium ratios in typical minerals range from 200:1 to 400:1. This metal may be produced in a secondary manner by the recycling of batteries, copper-cadmium alloys and powders from electric arc furnaces. It is estimated that the production of secondary Cd accounts for approximately 20% of the total production of metallic Cd. It is used in the manufacture of various products in common use, being the nickel-cadmium batteries the article that consumes most of the world’s production of this element (90%). A percentage of Cd is also used in the manufacture of pigments, coatings, stabilizers for plastics, non-ferrous alloys, electroplating, photovoltaic devices, etc. These activities related to the extraction of metal and the manufacture, use and disposal of Cd products, as well as agriculture, have the potential to release Cd into the environment and to be sources of exposure for humans and other animals [3,28].

The route of exposure impacts importantly Cd absorption: inhalation, that occurs mainly through tobacco smoke, approximates 25% (range 5–50%), whereas the oral route, through contaminated water and food (offal and seafood), was estimated at 5% (range 1–10%) [3].

Smoking one cigarette increases blood concentration by approximately 0.1–0.2 μg Cd per liter because each cigarette contains from 1 to 2 μg of Cd [1,3,29,30]; nevertheless, Cd content can vary depending on the origin of the tobacco leaves: in studies on Mexican cigarettes, it was found that each cigarette contained from 2.5 to 2.8 μg of Cd [31,32].

Considering a specific route of exposure for newborns, the presence of Cd in breast milk has been evidenced before [33,34] but recent findings once more highlighted the importance of this route since mouse pups showed a significant Cd exposure due to lactation after dams inhaled cadmium oxide (CdO) nanoparticles, opening the discussion about breast feeding as the major source of Cd exposure after birth and the clinical debate over lactation versus formula feeding in exposed populations [35].

Even if Cd concentrations in ambient air are generally considered as low [2], anthropogenic activities release large amounts of Cd, and air pollution also appears as a substantial source of exposure, especially in urban areas where the concentration of this metal ranges from 2 to 15 ng per cubic meter. However, some reports describe that Cd concentration in fine particulate matter with 2.5 micrometers or less in size (PM_2.5_) can be higher, for instance, in Mexico City and its metropolitan area, levels of 35 to 40 ng per cubic meter have been detected previously [3,36,37].

## 3. Cadmium Distribution during Pregnancy

During pregnancy, both inhalational and oral Cd absorption increase [35,38,39]. This effect can be explained by the physiological changes that take place during this stage, like increased respiratory rate, decreased gastrointestinal motility and decreased gastric emptying [40]. In addition, the overexpression of receptors and transporters in the gut due to high nutrient demand [41] can promote Cd absorption too. Cadmium accumulates in the lung or gut depending on the route of exposure; then, it is distributed to the liver, kidneys, placenta, mammary glands, uterus and fetus [42,43,44,45] and it can be excreted into the milk [46,47].

It is believed that the absorption of Cd in the intestine is facilitated by different transporters, such as the Divalent Metal Transporter-1 (DMT-1), calcium channels, amino acid transporters, and by endocytosis of the cadmium-metallothionein (CdMT) complex [19,48].

### 3.1. Role of Diet

The diet is an important source of Cd. A variety of foodstuff may contain important amounts of Cd, for example potatoes, wheat, seafood (mollusks and crustaceans), offal, spinach, cereal bars, chocolate, soy and grains [3,49]. Therefore, during pregnancy, diet is the main source of Cd exposure.

Nutritional status can influence the absorption and distribution of Cd. Deficiency of essential metals is related to an increase in oral Cd absorption of up to 10 times [50,51], because Cd uses different essential metal transporters as entry pathways. For instance, iron and/or calcium deficiency, result in remarkable increases of Cd accumulation in liver and kidneys [51].

On the opposite side, a diet high in essential micronutrients can decrease oral Cd absorption [52] or its toxic effects [53]. Antioxidants [54,55] and probiotics [56,57] have also been proposed to decrease Cd toxicity.

Thus, the influence of the nutritional status on increases of absorption and accumulation of Cd is evident so that malnutrition and gestation are risk factors for the toxic effects of Cd. If they occur in combination, the risk of the absorption and accumulation of Cd increases and therefore toxic effects may be potentiated.

### 3.2. The Role of DMT-1 in Cd Distribution during Pregnancy

The presence of the transporter DMT-1 in the gut is very important for iron absorption, and its expression is dependent of iron body levels [58,59]. Cadmium absorption increases when there is an iron deficiency, and this effect has been related with increased DMT-1 expression in the intestine [38,52,60].

During the gestational stage, there is a bigger iron demand because this metal is necessary for fetal development [61]. This necessary iron is obtained from iron stores, and in answer to depletion of iron stores during late pregnancy, the expression of DMT1 and ferroportin-1 (FPN1) increases in the gut to obtain iron from the diet [61]. Although FPN1 is not involved in Cd transport [62], Leazer et al. observed an increase of Cd absorption during late pregnancy that may be related to an increase of DMT-1 in this period [38]. The iron movement during gestation could be related to Cd movement too. In the late stage of pregnancy and lactation period, iron is transferred to the fetus and mammary glands to be secreted into the milk [61]. Nakamura et al. suggested a role for DMT1 in Cd transfer to the fetus [43] and it will be discussed in Section 4.

### 3.3. Role of Metallothionein in Cd Distribution

Metallothionein (MT) is a low molecular weight protein that is important for zinc and copper homeostasis. It is ubiquitous, and each MT molecule can bind seven atoms of Cd [63].

Brako et al. [47] using knockout (k.o.) mice for isoforms 1 and 2 of MT (MT-I/MT-II k.o.) observed that, during early pregnancy, Cd absorption and distribution is independent of MT. Meanwhile, during the late gestation and lactation stages, MT increases in the gut and restricts the movement of Cd to the blood but it does not inhibit total Cd absorption. Therefore, in the absence of MT (MT-I/MT-II k.o.) Cd concentrations in the blood, liver, placenta, mammary glands and fetus are higher than in normal mice [47].

On the other hand, MT is important for Cd redistribution during late pregnancy. Chan and Cherian (1993) exposed Sprague-Dawley female rats to eight daily injections (s.c.) of 1.0 mg Cd/kg as CdCl_2_ during 2 weeks. Then, 1.5 weeks later, the rats mated with an untreated male of the same strain. Later, the rats were sacrificed on gestational days (GD) 1, 7, 14 and a day before delivery. They observed that during the middle and late pregnancy, Cd liver concentrations decreased, and increased in the placenta and kidneys, meanwhile in non-pregnant rats the amount of Cd in liver and kidneys showed no apparent changes [64]. This Cd redistribution could be explained by MT redistribution, since they observed the same pattern with MT levels during this time of gestation [47,64]. Placenta has been showed to highly express megalin in cytotrophoblasts during first and third trimester [65]: megalin is a transmembrane receptor which mediates uptake and trafficking of many ligands including MT in kidney and other tissues [66,67,68]. However, other transporters that participate in receptor-mediated endocytosis of MT could be also involved [69,70]. These findings can be relevant for women in reproductive age who have been exposed chronically to Cd because this behavior increases the risk of Cd-induced toxicity in the kidneys and placenta during gestation.

## 4. Toxic Effects in Dams

There are many papers that evaluate the effects of Cd exposure during pregnancy, but most of them only assess toxic effects in the offspring and forget about dams. The toxic effects that have been observed in dams (Table 1) are dependent on Cd dose, exposure route, and the period of exposure i.e., before pregnancy, early pregnancy, late pregnancy and/or lactation period. However, Cd exposure during pregnancy has been associated with various effects on kidney, blood pressure and alterations of essential elements in the body, the latter being the cause of other pathologies in the mothers and fetuses.

### 4.1. Preeclampsia

Preeclampsia (PE) is a specific syndrome of gestation, characterized by increased blood pressure and proteinuria after the 20th week of gestation. The causes could be genetic, modifications in the vascular endothelium induced by lifestyle factors, or immunological disorders [83]. Using in vivo models, Cd exposure has been associated to preeclampsia development with increased systolic pressure and proteinuria [72,73,74]. The mechanisms suggested for preeclampsia induction by Cd exposure are: placental damage caused by oxidative DNA damage [65,72], and high levels of corticosterone in plasma, consequence of placental alterations (downregulation of 11β-hydroxysteroid dehydrogenase, 11β-HSD2) [73,75]. Other mechanisms possibly related to the development of preeclampsia are immunological disorders. Zhang et al. observed an increased activation of complement component 5 (C5) in preeclamptic patients, determined by the increase in the concentration of the fragment C5 (C5a) in blood samples, which is a marker for complement activation [74]. The complement activation has been considered to be partially responsible for the development of preeclampsia and kidney injury [84]. In the same study, Wistar rats were exposed to 0.125 mg of Cd/kg/day intraperitoneally from GD9–14, as a model of preeclampsia, which was confirmed by an increase in systolic blood pressure and proteinuria. With this model, they observed increased levels of C5a and angiotensin II type 1-receptor-agonist autoantibodies (AT1-AA) induced by Cd exposure. When the rats were treated with an antagonist of C5a receptor (0.5 mg of PMX53/kg i.p. starting from 1 day before Cd administration), the increases of pressure and protein excretion were minor. Moreover, losartan treatment prevented the Cd-induced increase in C5a levels. Thus, the authors suggested that Cd induce C5 activation through AT1-AA via AT1 receptor and therefore the preeclampsia characteristics [67,74].

However, all the studies linking Cd to preeclampsia have a disadvantage, they use a route of Cd exposure that is not environmentally relevant (intraperitoneal). Future studies with more relevant routes of Cd exposure are needed.

### 4.2. Kidney Damage

The kidneys are an important target of chronic Cd exposure [3]. During pregnancy, Cd is mobilized from the liver to the placenta and kidneys [64], also the expression of MT in the gut, favors the movement of Cd to the kidneys [47]. Therefore, kidneys are in great risk of experiencing Cd toxicity during pregnancy. Blum et al. exposed pregnant CD-1 mice to Cd by inhalation (CdO nanoparticles, 230 µg/m^3^) from GD4.5 to 16.5, and they observed the excretion of kidney injury molecule 1 (Kim-1) into the urine [35]. Kim-1 is an early biomarker of proximal tubule kidney injury that changes before creatinine [85], the most common biomarker for kidney damage. Chan and Cherian (1993), exposed rats to Cd two weeks before mating and observed kidney damage until lactation stage, described by an increase of blood urea nitrogen (BUN) [64]. In addition, changes in renal morphology have been reported, such as glomerular endotheliosis, infiltration of inflammatory cells [72], irregularly shaped nuclei and presence of vacuoles [35].

Due to the redistribution and increased Cd absorption during gestation, more studies are needed to assess renal damage in dams exposed to Cd.

### 4.3. Lesser Incidence of Pregnancy

Animal studies showed that Cd exposure during gestation prevented implantation or caused less implantation sites, more resorptions and less number of fetuses (see Section 6), which is translated into a lesser incidence of pregnancy [45,71,86]. During pregnancy, the uterus and the placenta are Cd targets. It is possible that Cd decreases implantation due to a disturbance of the activity of implantation-related enzymes: cathepsin-D and alkaline phosphatase [76]. Also, it can be due to Cd-induced blastocyst death after implantation, caused by decreased levels of progesterone triggered by a lesser number of trophoblast cells [45,76,77,87].

Cd is known as an endocrine disruptor [88]. As it will be stated in the subsequent section, progesterone and estrogen are necessary to maintain pregnancy. Cadmium decreases hormones levels during pregnancy, which in turn can be caused by a decrease of steroidogenic enzymes [76,77,78]. In addition, complement activation have been related to dysregulation of angiogenic factors and spontaneous abortion [89]. During pregnancy, Cd exposure increases C5a [74]. Higher maternal plasma concentration of C5a has been associated with miscarriage and fetal death [90,91]; therefore, the Cd-induced increase in C5a levels during pregnancy, could explain the lesser incidence of pregnancy; nevertheless, studies to clarify this relationship are necessary.

Even if hormone levels do not affect the maintenance of pregnancy [76], low doses of Cd may cause effects on the offspring (intrauterine exposure), which should be evaluated.

### 4.4. Altered Levels of Essential Elements in the Body

Since Cd is a divalent metal ion, it is absorbed using transporters for essential metal ions. During gestational stage, gastrointestinal absorption increases, and the transport of essential elements increases too. Also during this stage, Cd exposure has been associated with altered concentrations of micronutrients in internal organs. For instance, iron levels in liver, kidneys, placenta and fetus decrease [39,44]; zinc levels increase in liver and decrease in kidneys and placenta [44,64,92], and copper levels show contrasting behaviors in liver and kidneys [51,64]. Although there are differences in the route of Cd administration, all studies conclude that Cd induced lower micronutrient concentrations in the fetuses.

The alteration in the concentrations of essential metals could be explained by Cd competition for receptors and transporters in the gastrointestinal tract, thus decreasing the absorption of nutrients [51]. Similarly, the induction of proteins such as MT in liver, kidney and gut could increase the concentrations of metals, such as as zinc and copper [47,63]. In addition, Cd reduces the activity of enzymes such as ceruloplasmin during gestation [79]. Ceruloplasmin is an important protein for copper transport from the liver to other organs [93] and plays a crucial role in copper transport from the dam to the fetus [94,95]. This effect could explain the accumulation of copper in liver and/or kidney and its decreased distribution to the fetus.

### 4.5. Decreased Bone Mineral Density

Cd may influence bone turnover by disruption of calcium absorption due to competition for their transporters in the intestine, or as consequence of kidney injury [2,30]. There are many animal studies about Cd toxicity during pregnancy; however, almost none of them investigated calcium levels. Trottier et al. reported that Cd inhalation (50 μg/m^3^) during late gestation, decreased placental calcium levels after 5 days of exposure [80], suggesting that Cd interferes with calcium metabolism and/or transport. Another study showed that Cd promotes a loss of calcium from dam’s skeleton during gestation and lactation stages that was induced by a calcium deficient diet [81]. The known interference between Cd and calcium, in addition to the high calcium demand during gestation and lactation, suggests a risk of the mother and fetus to develop osteomalacia or osteoporosis due to Cd exposure but more studies are needed.

## 5. Transport of Cd across the Placenta

The placenta is the organ responsible for enabling the transference of nutrients from the mother to the developing fetus, as well as the secretion of hormones that play a major role in the maintenance of pregnancy. At some point, this organ was considered an effective barrier that protected the fetus from toxic agents, but now, it is well known that its capacity to block toxicants from reaching the fetus is somewhat limited.

In this regard, the expression of MT-I and MT-II in the placenta restrains the passage of Cd to the fetus [47,96]. For instance, the levels of Cd in pups of MT-I/MT-II knockout (k.o.) mice exposed to this metal in utero were 100-fold higher than wild type mice [47]. Nevertheless, although the placental expression of both isoforms of MT can be induced by oral Cd exposure [43], it seems that the ability of this protein to block its transfer from the dam to the fetus is partial or can be surpassed when large amounts of Cd are present, which leads to the diffusion of this metal across the placenta.

The molecular mechanism by which Cd reaches the developing fetus remains to be established. Some studies suggest the participation of metal transporters, such as DMT-1, Zrt/Irt-like protein 14 (ZIP-14) and zinc transporter 2 (ZnT2) [38,43].

The placental expression of DMT-1 increases in a time-dependent manner over the progression of gestation and is higher than in other dam organs like large and small intestine, liver, and kidney [38]. Additionally, the placental gene expression of metal transporters can be induced in Wistar rats by an oral exposure to increasing doses of Cd (0, 1, 2 and 5 mg Cd/kg) from 3 weeks before mating and until GD20 [43]. Taking this into account, and the fact that these transporters lack substrate specificity (their substrates include iron, manganese, cobalt and cadmium, among others), it is quite possible that they facilitate the diffusion of Cd across the placenta. Still, more studies are needed to fully comprehend the extent of participation of these proteins.

In addition to the aforementioned transporters, TRPV6 (transient receptor potential cation channel subfamily V member 6), also known as calcium transporter type 1 (CaT1), could play a role in Cd diffusion. A study by Moreau et al. [97] using primary cultures of human placenta showed its expression in cytotrophoblasts where it contributed to calcium uptake. This finding suggested its participation in calcium maternal-fetal transfer, which was later corroborated with TRPV6 k.o. mice, whose fetuses had lower serum calcium levels and mineral content [98]. TRPV6, however, is not specific for calcium uptake, in vitro studies have demonstrated its ability to transport other elements, such as zinc, lanthanum, gadolinium and Cd [99,100], which raises the possibility of this channel contributing to Cd passage across the placenta. Studies that approach this issue are still needed.

Similarly to TRPV6, it is possible that the membrane receptor megalin plays a role in Cd transfer. Megalin is known to participate in the reabsorption of the Cd-MT complex in proximal tubules [66] and it is present in cytotrophoblasts as well as syncytiotrophoblasts of human [65] and mice placenta [101] where it is thought to contribute to the transfer of nutrients to the fetus. So it is plausible that this receptor mediates, to some extent, Cd endocytosis and subsequent transport across the placenta; nevertheless, this speculation needs to be confirmed by experimental studies.

Moreover, the use of Cd in the core of nanoparticles with biomedical applications poses new challenges in terms of assessing Cd toxicity. Because of their small size, nanoparticles can reach further than larger compounds, so it is of primary importance to conduct studies that evaluate the rate and amount of placental accumulation and transfer of cadmium nanoparticles (Cd-NPs). Although some studies start to address this issue [87], there is a long way to go to understand the molecular mechanisms involved in the transfer of Cd-NPs.

### 5.1. Placental Susceptibility to Cd

The placenta is one of the primary targets of Cd toxicity. Placental effects of this metal have been described in several animal species as early as the mid-60s. Some of the effects reported by those early studies include placental necrosis, vascular congestion, hemorrhage, decreased utero-placental blood flow and leukocyte infiltration [102,103,104]. The progress in laboratory techniques in recent years has allowed the evaluation of Cd-induced structural and functional alterations in the placenta at a molecular level. Table 2 summarizes some of Cd placental effects reported in the contemporary literature.

The experimental designs used to assess Cd placental toxicity are very diverse. Most studies administered this metal by routes that are not relevant for human exposure, and those seem to have the most severe outcomes in placental structure and/or function, even at low doses. However, studies that used oral administration, which is the main route of exposure for the non-smoking population, also caused alterations that can affect placental functions and that, ultimately, could result in fetal damage [43,75,96,108].

For example, a higher rate of trophoblastic apoptosis/necrosis is the most reported result of Cd intoxication no matter the route, length or period of exposure [77,87,96,106,107]. Interestingly, this effect appears to be dependent on the cellular type, because the four types of trophoblasts have different sensitivities to Cd. Yamagishi et al. reported that this metal showed the highest affinity for cytotrophoblasts but the most severely injured cells were spongiotrophoblasts [107]. The reason for this, however, is not clear yet.

Trophoblastic death can lead, partially, to some of the other Cd effects, such as altered endocrine function because trophoblasts in the junctional zone are responsible for the synthesis of hormones like progesterone, estradiol and placental lactogens [76,77,87]. This, in turn, could affect the maintenance of the pregnant state.

The precise mechanism by which Cd exerts its harmful effects in the placenta has not been fully unraveled, but the high vulnerability of this organ may derive, at least in part, from the ability of the trophoblasts to synthetize MT, which leads to Cd accumulation. Also, recent evidence showed that intragastric exposure to Cd 20 days prior to mating, resulted in lower protein levels of the transporters ABCG2 and ABCB4 in the placenta. These transporters protect the fetus from xenobiotics by transferring them, against their concentration gradient, to the maternal circulation [109]. Thus, a lower presence of these molecules could also increase Cd accumulation in the placenta. Once accumulated, it can induce oxidative stress, DNA fragmentation and endoplasmic reticulum stress [77,87,106].

Knowing the importance of the placenta in the progression of pregnancy and the development of the fetus, placental toxicity plays a pivotal role in Cd fetal effects. Section 6 will discuss such outcomes.

### 5.2. Cadmium Distribution in the Fetus

Cadmium transfer to the fetus is limited, but once this metal has reached the fetal body, it is of great importance to know how it gets distributed because it gives us the opportunity to estimate the most vulnerable organs and the magnitude of its effects. Unfortunately, most studies measure Cd fetal burden as a whole [44,75,86,108,110] which can only tell us whether this metal reached the fetus or not.

Despite that, some authors show that Cd can build up in the liver and kidney of Wistar rat fetuses (GD20) and pups (PND1) after an oral exposure to 0.5 and 5 mg Cd/kg through gavage [34,96]. Cadmium levels in both organs were comparable in each experimental design, which suggests a similar expression of MT or other metal-binding proteins in fetal liver and kidney. Nevertheless, as postnatal development progresses, liver Cd concentration peaks around PND14, after which the levels start to decrease. Meanwhile, the kidneys start to accumulate this metal as time goes by. Altogether, these findings point at a redistribution of Cd from the liver to the kidney like the one that happens in adult individuals [34].

After an exposure through inhalation or a subcutaneous injection, Cd experiences a similar distribution. For instance, it accumulated in the liver, brain and heart of Hartley guinea pig fetuses exposed to a dose of 0.05 mg Cd/m^3^, for 4 h a day from GD35 to 40 [80]. Also, the exposure to 17.4 mg Cd/m^3^ for 2 h from GD8–20 significantly increased the levels of this metal in the kidney of Wistar rat fetuses [42]. Additionally, exposure to Cd acetate by a subcutaneous injection from 7 days prior to mating and until GD20 or PND21 also leads to hepatic Cd build up in rat fetuses and pups [111].

These studies suggest that Cd distribution in the fetal body is quite similar to that found in adult animals probably because the pattern of MT synthesis, distribution is alike [112]. More importantly, differences in the animal species, type of salt used, route and length of exposure do not seem to affect significantly its fetal distribution.

## 6. Effects of Cd in the Fetal Organism

Fetal effects of Cd intoxication have been known for many years and they span a wide variety of organs. Table 3 lists the main outcomes of Cd gestational exposure in the recent literature.

Like placental toxicity, the mechanism of fetal toxicity remains elusive. Multiple studies show contrasting evidence in terms of maternal transfer of Cd, which raises the question of whether fetal toxicity is consequence of Cd action per se or of placental toxicity. Because the mother-fetus binomial has a very fine balance and cannot be separated, it is most likely that toxic effects in the fetuses are the result of both Cd fetal distribution and accumulation, and placental alterations.

Placental changes play a critical role in prenatal toxicity because they modify its ability to produce hormones, as well as its function in oxygen and nutrient transfer. For instance, placental hormonal synthesis is not only of primary importance for pregnancy sustainment, but it can also have detrimental effects in dams and fetuses. Cadmium exposure of rats during the gestational period increases the levels of corticosterone in the placenta and plasma of dams and fetuses possibly due to a decreased activity of placental 11β-HSD2 [73,75]. High cortisone levels have been related to the presence intrauterine growth restriction (IUGR), which, at the same time, can induce perinatal morbidity and the development of certain diseases in adult life due to fetal programming [75].

In addition, Cd-induced deficiency in the maternal transfer of nutrients like glucose, and essential elements, such as copper, calcium, sodium, potassium, zinc and iron [44,80,108,111,123], which are necessary for an optimal development, resulted in reduced body weight and fetal death of the offspring of rats and guinea pigs and are two of the most reported effects of this heavy metal (Table 3). This reduced transfer may be due to a decrease in the protein levels of transporters [108] and competition between Cd and other metals for their transporters in both the intestine and placenta of pregnant rats. In addition, the reduction in the lumen of the blood vessels in the labyrinth region of the placenta (Table 2) can interfere with nutrient transfer because the area of exchange is significantly smaller.

### 6.1. Teratogenicity

Cd is a well-established teratogenic agent. Its effects were reported 30 years ago, and even though the type of effects may vary depending on the dose, route and length of exposure, they have been reproduced by many groups, which underlines the danger of Cd gestational exposure and stresses the importance of finding ways to counteract those outcomes. In this sense, some studies found that co-exposure with the amino acid glycine can revert Cd-induced malformations due to the antioxidant activity of this molecule (evidenced by a decrease in lipid peroxidation levels) [86,119].

The main teratogenic effects caused by Cd are summarized in Table 3 and they include cleft palate, tail deformity, delayed chondrogenesis and postaxial forelimb ectrodactyly. The mechanism of toxicity behind these outcomes is not entirely deciphered, although some studies have shed some light on some of them. For example, postaxial forelimb ectrodactyly can be the result of a higher rate of apoptosis in limb buds, decreased expression of molecules involved in limb patterning, such as Fgf4, and decreased polarizing activity of the limb bud [118,121]. Interestingly, this outcome shows a right limb predominance when the exposure occurs on GD9, the reason why this happens is unknown. Also, altered chondrogenesis may be caused by a lower expression of Sox9 in the zeugopods of mouse limb buds [118].

### 6.2. Central Nervous System

Neural tube defects comprise a series of congenital diseases that affect the brain, spine and spinal cord. Their etiology is poorly understood, but it is thought that genetic, nutritional and environmental factors, or a combination of all, can induce them [124]. In this regard, when administered in the early organogenesis (during neurulation), Cd can induce open neural tubes that can result in anencephaly, exencephaly and encephalomeningocele (Table 3). Considering that folic acid is a vitamin necessary for a normal development, these Cd-induced defects may be the result of a lower folate content in embryos, triggered by a decreased expression of folate transporters in the placenta [45]. Additionally, direct actions of this metal may also contribute since the ex vivo exposure of mice embryos to 1 μM of CdCl_2_ for 48 h caused a higher incidence of open neural tubes. This outcome correlated with the presence of oxidative stress that was counteracted by glycine co-exposure [119]. Furthermore, evidence shows that Cd can alter the expression of between 20 and 30 genes involved in CNS development in two strains of mice (Swiss and C57BL/6J) as early as 12 h after exposure. More importantly, a higher rate of cases of mice with exencephaly suggested that the C57BL/6J strain has a higher sensitivity to the action of this metal, although the reason for this is not known [113].

In the late organogenesis, Cd can also cross the blood-brain barrier and accumulate in the brain of the offspring after a single exposure [80]. Once there, it can cause brain edema [87] and, possibly, long-term effects.

### 6.3. Liver

The liver is one of the main organs where Cd builds up and as such, is the target of its toxicity. The exposure of Wistar rats to drinking water containing 10 mg/kg CdCl_2_ during gestation causes degenerative changes in hepatocytes of fetuses at term, such as dilation of the smooth endoplasmic reticulum, slightly damaged nuclear membranes, and mitochondrial dilation [125]. In addition, a higher dose of Cd (50 ppm CdCl_2_) changes the hepatic mRNA and protein levels of the glucocorticoid receptor due to modifications of the level of methylation on its promoter region. These alterations changed the expression of phosphoenolpyruvate carboxykinase and acyl-CoA oxidase that are involved in carbohydrate and lipid metabolism, respectively. Interestingly, all modifications found were sex-dependent [116], which suggests that Cd has a differential mechanism of toxicity.

Similarly, subcutaneous administration of Cd decreases the activity of estradiol metabolizing enzymes in fetuses, and 21-day old rat pups [111].

### 6.4. Kidney

The deleterious effects of Cd in the kidney of adult individuals, and the molecular mechanisms behind them are the subject of a vast amount of studies. The fetal kidney as a target of this metal, however, is far understudied in comparison.

Cd can reach and accumulate in fetal kidney [34,42,96]; thus, it can cause structural alterations and damage just like in fully developed kidneys. Accordingly, Roman et al. conducted a study that showed that a single intraperitoneal injection on GD10 with 5 mg CdCl_2_/kg induces significant alterations in proximal and distal tubules, as well as glomerular edema in the offspring [126]. More recently, our group found that inhalational exposure to Cd from GD8 to GD20 provoked tubular necrosis and degeneration, among other effects, in the kidneys of Wistar rat fetuses (GD21). Such alterations correlated with an increase in the levels of early kidney injury biomarkers in amniotic fluid samples that are composed of fetal urine [42]. Similarly, a previous study presented evidence that repeated doses of Cd increased β2-microglobulin excretion and lower activity of γ-glutamyl transferase, alkaline phosphatase and N-acetylglucosaminidase in the urine of 3-day old rat pups, which suggested a reduced number of nephrons or a delayed tubular maturation [115]. All these renal outcomes could be the result of Cd-induced oxidative stress caused by lower levels of glutathione (GSH), superoxide dismutase (SOD) and catalase (CAT) [110]; nevertheless, further studies are required to corroborate these conclusions.

In addition, there is increasing evidence that links low birth weight with low nephron endowment [127]. Therefore, it is plausible that even if Cd does not reach fetal kidneys, it can cause low nephron endowment with subsequent alterations of renal structure and function, such as glomerulosclerosis.

Lastly, whether the renal effects caused by Cd gestational exposure are permanent or not, is still on debate because there is contrasting evidence that supports either possibility [34,115]. Discrepancies found may be the result of differences in the experimental models used, such as the period and length of exposure. Therefore, it is of great importance to keep on studying the renal effects of an in utero exposure to Cd since this organ is particularly vulnerable to this heavy metal as a result of its long embryonic development and its ability to accumulate Cd.

## 7. Conclusions

**Figure 1:**
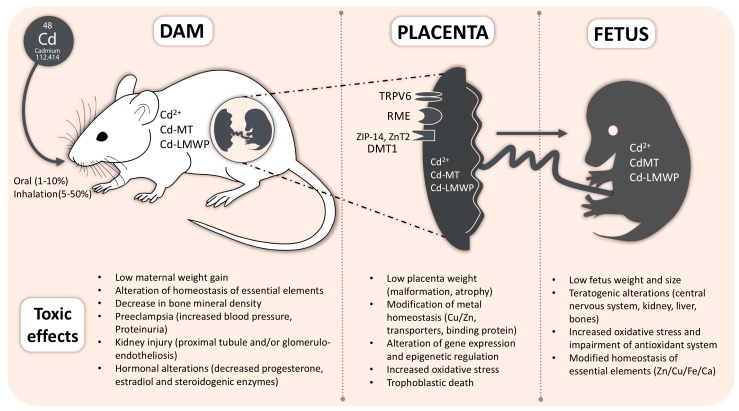
General scheme of the toxic effects of Cadmium (Cd) exposure in dam, placenta and fetus. MT: Metallothionein; LWMP: Low Molecular Weight Proteins; RME: Receptor-Mediated Endocytosis (e.g., Megalin or 24p3 Receptor); TRPV6: Transient Receptor Potential Cation Channel Subfamily V Member 6; DMT1: Divalent Metal Transporter-1; ZIP-14: Zrt/Irt-like Protein 14; ZnT2: Zinc Transporter 2.

This review of the current literature illustrates how exposure to Cd during pregnancy deeply impacts the health of the fetuses and mothers and underlines the complexity of the multi-organs toxicity induced by this heavy metal (Figure 1).

In addition, it is important to highlight that many facets of Cd toxicity remain unclear and that some dogmas related to the physiological handling of Cd are still discussed and need to be clarified. For example, our vision of the central role of metallothionein as the main carrier of Cd in blood stream or as the main source of delivery of Cd to proximal tubule epithelial cells (through receptor mediated endocytosis), could change in the coming years due to the evidence of the major role of other specific metal-binding proteins, such as transferrin, ferritin and Neutrophil gelatinase-associated lipocalin (NGAL), or new receptors, such as Lipocalin-2 (24p3/NGAL), involved in its binding and transport as well as the recent discovery of novel entry pathways for Cd [4,69,128].

## Figures and Tables

**Table 1:** Cadmium toxicity in dams.

Outcome	Reference
Lower maternal weight gain.	[45,71]
Increase of systolic blood pressure and increased proteinuria associated with preeclampsia.	[72,73,74]
Abnormal glucocorticoid synthesis; induction of angiotensin II type 1-receptor-agonist autoantibodies (AT1-AA) and activation of complement component 5 (C5).	[73,74,75]
Kidney injury reported by excretion of Kim-1 into urine; increase of blood urea nitrogen; changes in the morphology of proximal tubules, like irregularly shaped nuclei and presence of vacuoles. Glomeruloendotheliosis and infiltrated inflammatory cells.	[2,29,52]
Decrease of progesterone and estradiol in placenta and plasma and decreased steroidogenic enzymes in reproductive organs. Increased uterine weight.	[45,76,77,78]
Alteration in the levels of essential elements in the body.	[39,43,44,79,80]
Decreased bone mineral density.	[81,82]

**Table 2:** Cadmium toxicity in placenta.

Outcome	Reference
Reduction of placental weight.	[77,87,105]
Decreased levels of total proteins, RNA, total lipids, cholesterol and glycogen.	[76]
Altered mRNA and protein levels, as well as activity of steroidogenic enzymes: Decreased: 3β-Hydroxysteroid dehydrogenase (HSD), 17β-HSD and 11β-HSD2. Increased: CYP11A1, CYP11B1 and CYP21.	[73,76]
Altered production of hormones necessary for the maintenance of pregnancy.	[76,77,87]
Placental atrophy, and swelling, vacuolization, deformation and death of trophoblasts due to apoptosis and necrosis in the junctional and labyrinthine zones of the placenta. Increased mRNA levels of molecules involved in apoptosis (p.53 and Bax).	[77,87,96,106,107]
DNA fragmentation in the junctional zone.	[77]
Decreased Zn and Cu placental concentration and altered metal transporters expression.	[38,43,87,96]
Altered MT mRNA and protein expression.	[43,107]
Oxidative stress and endoplasmic reticulum stress.	[87,106]
Reduced inner space of maternal and fetal blood vessels in the labyrinth layer. Thickening in the media vessel walls.	[73,105,106]
Higher corticosterone levels.	[73,75]
Decreased mRNA and protein levels of glucose transporter 3 (GLUT3). Hypermethylation of GLUT3 promoter region (E19.5).	[108]
Increased placental mRNA and protein levels of DNA methyltransferase 3-like (DNMT3L) and DNA methyltransferase 3 β (DNMT3B).	[108]
Lower protein levels of ATP-binding cassette (ABC) ABCG2 and ABCB4 transporters.	[109]
Decreased mRNA and protein levels of placental proton-coupled folate transporter (PCFT).	[105]

**Table 3:** Cadmium toxicity in fetuses and pups.

Outcome	Reference
Lower fetal body weight, length and head diameter.	[42,73,75,77,87,105,106,108,110,113,114,115,116]
Higher number of resorptions, dead fetuses and post-implantation losses.	[77,86,87,105,107,113,117]
Increased apoptosis and decreased proliferation in the mesenchyme of limb buds of embryos.	[118]
Embryos with lower morphological score, somites number, DNA content, yolk sac diameter and cephalic length.	[119]
Higher percentage of embryos with an open neural tube and altered expression of genes related to the development of the Central Nervous System (CNS) and cell cycle arrest.	[113,119]
Increased levels of malondialdehyde (MDA) and myeloperoxidase (MPO) and decreased levels of Glutathione (GSH), Superoxide dismutase (SOD) and catalase (CAT) in embryos, placenta, fetal kidneys (except MPO), and fetal liver.	[86,110,119]
Fetal symmetrical kidneys, and renal cavitation and damage (tubular necrosis and degeneration, presence of hyaline cylinders in tubules, and proteinaceous material in the renal pelvis).	[42,120]
Delayed chondrogenesis that leads to decreased ossification of head bones, sternebrae and cervical vertebrae. Further, Cd causes fused ribs and vertebrae. Absence or lesser number of vertebrae, skull bones, ribs, tail bones, metacarpal and metatarsal bones, and phalanges.	[86,117,118]
Increased frequency of abnormalities such as cleft palate, unilateral anophtalmia, microphtalmia, hypoplasic lungs, genital anomalies, vein deformation, postaxial forelimb ectrodactyly (predominantly right-sided and with the loss of digit 5), clubfoot, polydactyly, anencephaly, exencephaly, encephalomeningocele, micrognathia, exophthalmos, tail deformity, amelia, brachygnathia, omphalocele, anotia, hemoperitoneum, brain edema and undifferentiated limbs.	[86,87,105,106,113,117,118,120,121]
Higher Cd levels in blood, liver and kidneys in the offspring at postnatal days (PND) 0 through 60.	[34,42,80,96,111,122]
Diminished weight gain of the offspring from PND0 to 21.	[122]
Lower concentration of Zn, Fe and Cu in fetal liver, and Ca levels in fetal kidney.	[111,123]
Lower activities of hepatic estradiol metabolizing enzymes (17-β-hydroxysteroid and UDP glucoronyl transferase) in fetuses (GD20) and pups (PND21).	[111]
Decreased DNA and glycogen hepatic content at PND21.	[111]
At PND21, lower activities of alkaline phosphatase, acid phosphatase and Na^+^/K^+^ ATPase in kidney tissue.	[122]
Reduced anogenital index in pups at PND1 and 21.	[114]
Delayed hair appearance, testicular descent, palmar grasp and negative geotaxis in pups.	[114]
